# Graft Rejection Rate and Graft Failure Rate of Penetrating Keratoplasty (PKP) vs Lamellar Procedures: A Systematic Review

**DOI:** 10.1371/journal.pone.0119934

**Published:** 2015-03-17

**Authors:** Zarique Z. Akanda, Abdul Naeem, Elizabeth Russell, Jillian Belrose, Francie F. Si, William G. Hodge

**Affiliations:** 1 Western University, London, Ontario, Canada; 2 St. Joseph Health Care (Medical Librarian), London, Ontario, Canada; 3 Western University, Ivey Eye Institute, London, Ontario, Canada; University of Louisville, UNITED STATES

## Abstract

**Purpose:**

The aim of our investigation was to conduct a quantitative meta-analysis of the present world literature comparing the major surgical outcomes of penetrating keratoplasty (PKP) to lamellar procedures. Our goal is that clinicians, eye bank administrators, and health policy makers will be able to utilize this study in implementing decisions in regards to corneal transplantation.

**Methods:**

Pooled measures of association were with odds ratios and because of study heterogeneity, the pooled effects were assumed to follow a random effects model (DerSimonian-Laird). The comparisons were between 1) PKP’s and all lamellar procedures (anterior AND posterior) and then 2) between PKP’s and all anterior lamellar procedures and 3) PKP and all posterior lamellar procedures.

**Results:**

For PKP vs anterior lamellar procedures, the pooled odds ratio for rejection of PKP over lamellar keratoplasty (LK) was 3.56 (95% CI: 1.76-7.20) and for outright failure, the pooled odds ratio of PKP failure vs LK was 2.85 (95% CI: 0.84-9.66). For posterior lamellar procedures, the pooled odds ratio for rejection of PKP over LK was 1.52 (95% CI: 1.00-2.32). The pooled odds ratio for outright failure of PKP over posterior lamellar procedures was 2.09 (95% CI: 0.57-7.59). The follow up time was significantly longer for full transplants than for lamellar procedures.

**Conclusions:**

For both anterior and posterior lamellar procedures, the odds ratios comparing rejection of full transplants to lamellar procedures (both anterior and posterior individually) were significantly higher in the PKP group. For outright failure, the PKP group also had a higher risk of failure than the lamellar groups but this was not statistically significant in either instance (anterior or posterior). Some of the clinical differences benefitting lamellar procedures may at least be partly explained by follow up time differences between groups and this needs to be accounted for more rigorously in future studies.

## Introduction

The multi layered cornea tissue is being exploited by modern cornea surgeons based on the anatomic diversity of its several layers [1]. When the health of the cornea is compromised and medical intervention is insufficient, such as shape irregularities, scarring, and corneal dystrophies, surgical intervention in the form of corneal transplantation (keratoplasty) is required [2]. For over the greater part of a century, the surgical technique of choice has been penetrating keratoplasty (PKP) [3], in which all five layers of the cornea are removed and replaced with donor tissue [4]. Some of the complications of performing PKP include unpredictable astigmatism, slow rehabilitation, and prolonged use of topical steroids and associated side effects of chronic topical steroid use [5]. Critically, immunological rejection of the donor cornea’s endothelial cells may occur. This may lead to graft failure and endothelial cell attrition following surgery [6]. In the past decade there has been an increasing trend in much of the world toward using novel keratoplasty techniques known as modern lamellar keratoplasty (LK); it has become the technique of choice in the UK, and almost half of all keratoplasty procedures are now lamellar in the USA [7]. LK potentially addresses the issue of immunological incompatibilities between host and donor tissue (at least in part), as diseased layers of the cornea are specifically removed and healthy tissue is preserved [8]. Sub branches of these keratoplasties include:

- Deep anterior lamellar keratoplasty (DALK) or pre-descemet anterior LK which removes the stroma down to the descemet’s membrane for individuals experiencing diseases where the stroma is compromised [9]. In this study, these procedures are referred to as “anterior lamellar procedures”.
- Descemet's Stripping Endothelial Keratoplasty (DSEK) and Descemet's Membrane Endothelial Keratoplasty (DMEK) refer to the replacement of recipient endothelium with the donor endothelium; DSEK provides stroma whereas DMEK does not [10]. In this study, we call these procedures will be referred to as “posterior LK”.

Advantages of LK include faster recovery time, reduced astigmatism, and minimized endothelial cell loss [11]; however, lamellar techniques are technically demanding, resulting in prolonged surgical time, and sometimes produce suboptimal or only equal visual results in comparison to PKP [12]. Although LK procedures are rapidly gaining ground as the procedures of choice, there is still conflicting evidence comparing the benefits of each LK to PKP [13]. Reviews thus far have compared the procedures qualitatively. There is now enough literature available to perform a quantitative high quality review comparing the procedures from analytic studies. The aim of our investigation was to conduct a quantitative meta-analysis of the present world literature comparing the major surgical outcomes of PKP to lamellar procedures. We hope that clinicians, eye bank administrators, and health policy makers will be able to utilize this study in implementing decisions in regards to corneal transplantation.

## Methods

The aim of this investigation was to compare both graft rejection and graft failure between full cornea transplant and both anterior and posterior lamellar procedures. The research question and keywords were defined in consultation with a technical expert panel and then the search was performed by a library information specialist.

### Literature Search Strategy

A thorough search strategy was implemented to produce the highest return of relevant clinical studies. The strategy was devised with ophthalmologists and information specialists.

Literature was obtained from an exhaustive search from the following databases: Cochrane, Embase, Medline, and PubMed. After a bibliographic record was obtained, duplicates were removed with the citation software EndNote. Key words that formed the basis of the search were “penetrating keratoplasty”, “lamellar keratoplasty”, “immunologic rejection”, “graft failure” and synonyms. Furthermore, grey literature was retrieved from special databases, conferences and seminars, and from professional associations. These included the American Academy of Ophthalmology Annual Meeting, the Association for Research in Vision and Ophthalmology (ARVO), the Canadian Ophthalmology Annual Meeting and the European Ophthalmology Society Meetings. References from publications which passed the first level of screening were manually searched to acquire further unique resources. The search was initially performed in June 2013 and monthly updates were done until March 2014.

### Eligibility Criteria

Published and unpublished studies from 2000 onwards were eligible for inclusion; no restriction was placed on the language or type of publication. Only analytic studies were included that compared PKP to a lamellar procedure (randomized trials, cohort studies). Case series without a comparator were not included. Additionally, a publication was considered relevant if the study looked at human populations of age 18 and over. In order to maximize the yield of publications, there were no criteria for specific reasons for receiving surgery. Studies had to look specifically at penetrating and LK in relation to the primary outcomes of graft rejection and graft failure. Graft rejection was defined as at a minimum: anterior chamber inflammation at least one month after the surgical procedure. Graft failure was defined as irreversible cornea edema or opacity. Secondary outcomes were included when presented in the publication but were not necessary for inclusion. They included: visual acuity, endothelial cell density, rehabilitation times, and surgical complications. Studies looking at artificial corneas and stem cell transplants were excluded. Studies with a sample size of less than 20 were also excluded.

### Selection Method and Data Extraction

Relevant bibliographic entries were entered into the internet based systematic review software EPPI reviewer version 4. The software directly recorded the evaluation of each publication, and provided a comparison from multiple reviewers. These results were used to determine eligibility of each study based on defined selection criteria. The rationale behind exclusion of ineligible publications was also recorded.

Two levels of screening were undertaken in selecting papers. Level 1 screening determined the eligibility of the publication from the title and abstract. If the publication was deemed eligible, it would proceed to level 2 screening where a full review of the text was carried out. Evaluations of publications in each of the screening processes were carried out by two reviewers. In cases where there was disagreement, a conclusion was reached by consensus, or a third party if necessary.

### Study Quality

The quality of studies was independently assessed by two evaluators. All studies were evaluated using the Down’s and Black quality assessment scale. The range of possible values are 0–32 with scores <15 considered poor, 15–19 as fair, and >20 as good quality.

### Data Synthesis

In cases where the data was sufficiently homogenous, a meta-analysis was performed for the defined outcomes. A pooled odds ratio was calculated to compare the association between groups. Due to study heterogeneity, the pooled effects were assumed to follow a random effects model (DerSimonian-Laird). The comparisons were between 1) PKP’s and all lamellar procedures (anterior AND posterior) and then 2) between PKP’s and all anterior lamellar procedures and 3) PKP and all posterior lamellar procedures.

The Begg test was used to check for publication bias after graphing the potential bias via Funnel plots. Meta-regressions were performed for study level and clinical level variables to see if confounders had influenced the outcomes studied. Multiple comparisons in the meta-regression were adjusted by using a Monte Carlo simulation of 20,000 trials. This random permutation can be used to adjust for multiple testing by comparing the calculated t-statistic for every covariate with the largest t statistic for any covariate in each permutation under Ho that all regression coefficients are zero. All statistical calculations were done using Stata 13 software (College Station, Texas).

## Results

**Fig 1:**
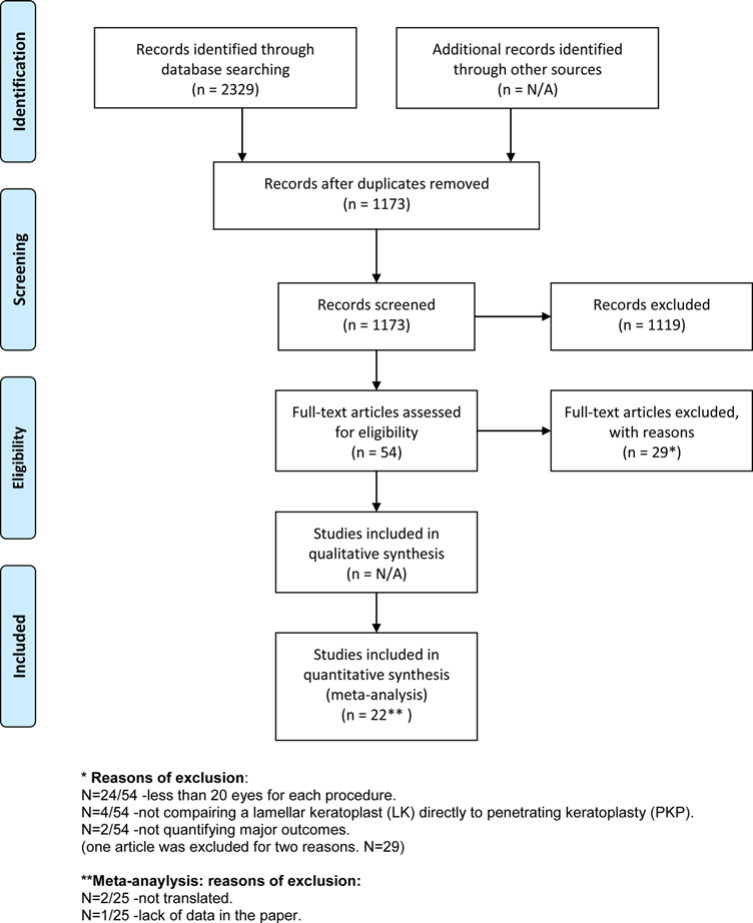
Prisma Diagram. The flow chart of the systematic review process.

The PRISMA diagram for this review is shown in Fig. 1. The final review included 22 papers [14–35]. Details of the 22 papers were summarized in S1 Dataset. Three of these papers studied PKP vs lamellar keratoplasty but did not report graft rejection or failure (they reported on cornea sensation-1 paper and visual acuity-2 papers). Three studies were randomized clinical trials and the remaining 19 studies were cohort studies. The sample sizes ranged from N = 38 to N = 1370 with a mean sample size of N = 271. The mean age in the PKP group and the lamellar group were both 55.3 years. 50% of the full PKP group were female while 53% of the lamellar group were female. The mean follow up for the PKP group was 30.4 months and 21.4 months for the lamellar group (p = 0.04). The mean initial logMAR vision for the PKP group was 0.64 and was 0.58 for the lamellar group (*p* = 0.51). The average quality assessment of the studies was on the high range of “fair” (mean 18.2, range of 15 to 21). Topical steroid use occurred for 87% of the duration of follow up in the PKP group and 82% of the duration in the lamellar group. Oral steroid use was needed in 12% of the PKP group and 8% of the lamellar group with an average dose of 40 mg in the PKP group and 32 mg in the lamellar group. Fig. 1 shows the flow chart of the systematic review process.

**Fig 2:**
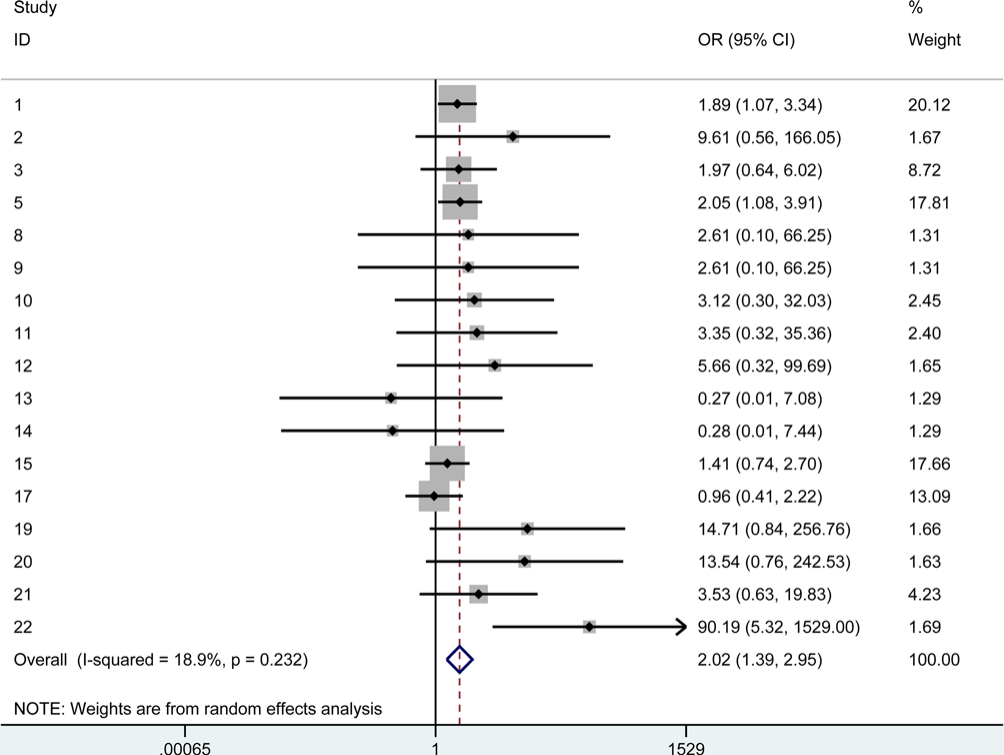
The summary Forest plots of the pooled odds ratio for PKP rejection over lamellar rejection.

**Fig 3:**
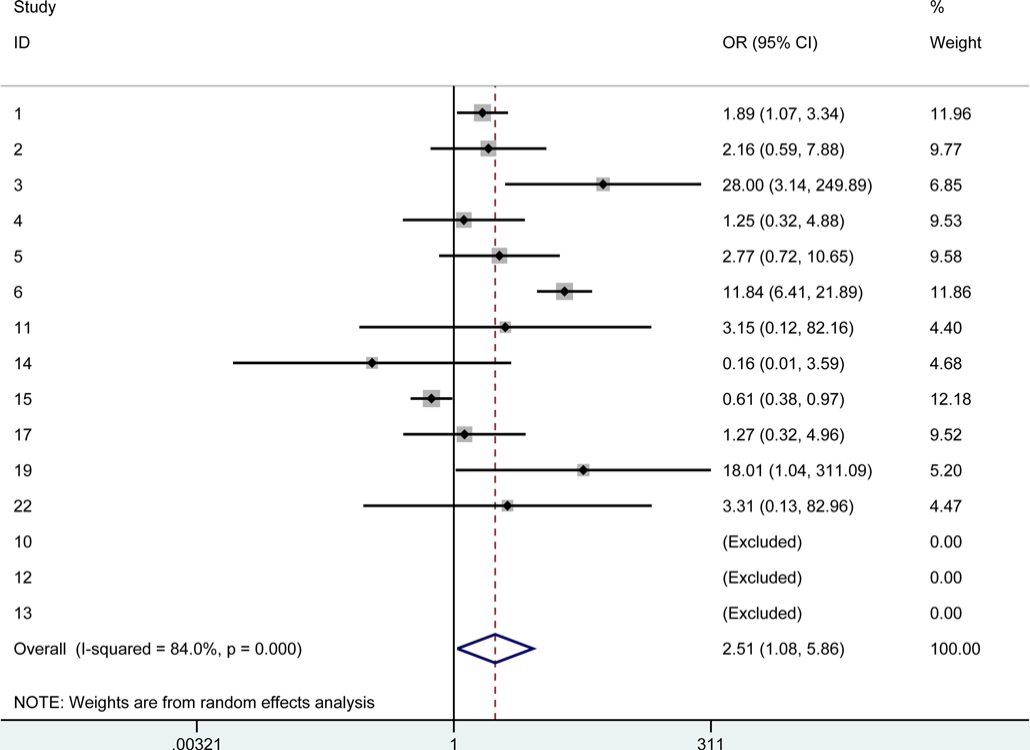
The summary Forest plots of the pooled odds ratio for PKP failure over lamellar failure.

We first compared the rejection rate and failure rate of PKP vs all lamellar procedures. The pooled odds ratio for PKP rejection over lamellar rejection was 2.02 (95% CI: 1.39–2.95). The pooled odds ratio for PKP failure was 2.51 (95% CI 1.08–5.86). The summary Forest plots for these outcomes are shown in Fig. 2 and Fig. 3. Fig. 2 shows the pooled odds ratio for PKP rejection over lamellar rejection and Fig. 3 the pooled odds ratio for PKP failure over lamellar failure.

Because anterior and posterior lamellar procedures are fundamentally different operations, we subdivided the analysis into PKP vs anterior lamellar procedures and then posterior lamellar procedures. For PKP vs anterior lamellar procedures (12 papers), the pooled odds ratio for rejection of PKP over LK was 3.56 (95% CI: 1.76–7.20). For outright failure, and the pooled odds ratio of PKP failure vs LK was 2.85 (95% CI: 0.84–9.66). For posterior lamellar procedures (7 papers), the pooled odds ratio for rejection of PKP over LK was 1.52 (95% CI: 1.00–2.32). The pooled odds ratio for outright failure of PKP over posterior lamellar procedures was 2.09 (95% CI: 0.57–7.59).

Because confounders may influence our results, we performed meta-regression on all three datasets (Table 1). Given that age, gender, and visual acuity were similar in each group, it was not surprising that none of these variables were significant predictors of any of our outcomes. We checked for topical steroid dose and duration on our outcomes and neither were significant. We further tested study type and sample size (recruited and completed) on our outcomes and none were significant predictors of the outcomes. Table 1 shows meta-regression summary for covariates on rates of cornea rejection.

**Table 1:** Meta-regression Summary for Covariates on Rates of Cornea Rejection.

Variable	Coefficient	Standard Error	P Value	95% CI
**Sample Size**	-.0009	0.004	0.83	(-.01, 0.009)
**Study Design**	4.18	1.95	0.21	(-0.24, 8.60)
**Percent Female**	-1.36	0.15	0.07	(-3.25,0.53)
**Mean Age (decade)**	-1.58	0.99	0.17	(-4.02, 0.863)
**Follow Up (months)**	2.61	1.06	0.49	(-1.21,5.19)
**Visual Acuity**	0.72	0.36	0.81	(-3.21, 4.22)
**Steroid Dose (Oral)**	1.33	0.56	0.61	(-1.35, 3.16)
**Steroid Duration (Topical)**	1.44	0.51	0.44	(-1.35, 3.21)

(Response Variable Log Odds of Rejection)

Importantly, follow up time was significantly different between PKP and lamellar groups and time to failure analyses were rarely performed. Because PKP's were followed on average 9 more months than lamellar patients, this may have been an important contributor to the results. Longer follow up may explain some of the increased rejection and failures in PKP patients. In our meta-regressions, we found that for PKP vs anterior lamellar rejections, the follow up times of the anterior LK group were indeed significantly shorter than the PKP group (p = 0.05). However when we controlled for multiple comparisons with a Monte Carlo simulation, this difference lost statistical significance (p = 0.18). When we looked at meta-regressions for PKP vs anterior LK failures the follow up times between groups were not significantly different in both uncorrected (p = 0.52) and Monte Carlo corrected (p = 0.56) meta-regressions. For PKP vs posterior lamellar grafts, both uncorrected (p = 0.28) and Monte Carlo corrected metaregressions (p = 0.42) showed no significant contributions of follow up time to the comparison of rejection rates. For outright failure the difference in follow up was significant in the unadjusted metaregression (p = 0.03), but not in the Monte Carlo adjusted metaregression (p = 0.11).

For all comparisons performed, the Begg test for publication bias was not statistically significant.

## Discussion

The odds ratios comparing rejection of full transplants to lamellar procedures (both anterior and posterior individually) were significantly higher in the PKP group. For outright failure, the PKP group also had a higher risk of failure than the lamellar groups but this was not statistically significant when an appropriate random effects meta-analysis was used in either instance (anterior or posterior). When we pooled all lamellar procedures together and compared them to PKP’s, PKP’s had a statistically significant higher rate of both rejection and failure. This pooling of all lamellar procedures allowed for increased power but it can be argued that pooling the results of anterior and posterior LK is not biologically wise as the operations are so different.

Plausible explanations for higher rejection rates in PKP over lamellar grafts include lack of donor endothelial cells (anterior lamellar grafts) as well as transplanting less tissue (anterior and posterior lamellar grafts) and performing a less traumatic and thus less inflammatory prone procedure (anterior and posterior lamellar grafts) [36].

We performed meta-regression to study whether any study related or clinically related variables influenced these results. For study size and type, age, gender and initial visual acuity, there was no effect on the results.

However using our meta-regression techniques, a small effect was found from length of follow up and this may be important clinically. Patients with PKP’s were followed longer than lamellar procedure patients and this may at least partly explain the results that PKP patients had higher rates of rejection and failure. The longer length of follow-up allows for a greater amount of time in which these outcomes may develop and be observed in patients. One can envision this happening especially early in the history of lamellar procedures. Full PKP’s were being practiced by virtually all cornea surgeons thus follow up could be done in a local practice and would likely to be complete. In contrast, lamellar procedures were being done by only some cornea surgeons by referral, who would then send the patients’ back to the original ophthalmologist, sometimes at far geographic distances. This could result in follow up times for lamellar procedures being more incomplete (and thus shorter) than for full grafts.

When we analyzed this issue in more detail we used similar techniques as in most statistical analyses. First we looked at the effect of follow up time on the results in a single test but performed this for many variables (as outlined above). These results showed a statistically significant effect of follow up time on PKP vs anterior LK rejections and on PKP vs posterior lamellar failures. However as in all statistical tests that use multiple comparisons, it is most appropriate to adjust for these multiple comparisons. When we did so with Monte Carlo simulation, the statistical significance was lost.

Multiple comparison adjustments are always a philosophical issue. In general, they are wise to do, but when one variable clearly has the most potential to influence results as in our study (follow up time), it can be argued that the multiple comparison is unnecessary and even reduces power.

When we tried to adjust our results by including studies of only similar follow up time, we had a significantly reduced number of studies available and our power was too low to make meaningful conclusions.

Hence our conclusion is that PKP has a higher rate of both rejection and outright failure than lamellar procedures, and statistically significantly so with rejection. However, when we looked for explanations for this effect, at least a small part of this effect is explained by follow up time differences between groups and this may be clinically significant. In future comparative studies of these procedures, surgeons should ensure that follow up time is as similar as possible between groups or that time to failure analyses are performed as some of the benefits of lamellar procedures over PKP may be explained by this difference in follow up time.

## Supporting Information

S1 Dataset Details of the 22 papers. (XLSX)

S1 PRISMA Checklist For reviewers. (DOC)
